# Complete Genome Sequence of Lacticaseibacillus paracasei Strain NSMJ15

**DOI:** 10.1128/MRA.01030-21

**Published:** 2021-12-09

**Authors:** Ji Young Jung, Kook-il Han, Hyun Mi Jin, Young Ho Nam, Hye Kyeong Kang, Mi Hwa Lee

**Affiliations:** a Microbial Research Department, Nakdonggang National Institute of Biological Resources (NNIBR), Sangju-si, Gyeongsangbuk-do, South Korea; University of Delaware

## Abstract

We report here the complete genome sequence of Lacticaseibacillus paracasei NSMJ15, isolated from makgeolli (a traditional Korean fermented liquor) and shown to have potentially probiotic characteristics. The genome consisted of a 2.79-Mbp chromosome contig and four plasmids having a total of 2,947 genes, including 2,690 coding sequences.

## ANNOUNCEMENT

Probiotics are generally considered a means to restore the balance of gut microbes and promote beneficial functions of the gut (1, 2). Lactic acid bacteria (LAB) are a major group of probiotic microorganisms in both humans and animals and are being extensively investigated in studies on probiotics (1–4). Lacticaseibacillus paracasei (formerly “Lactobacillus paracasei”) strain NSMJ15 was isolated from makgeolli, a traditional Korean fermented liquor, and *in vitro* study showed its probiotic potential, in particular, its antimicrobial properties against gut pathogens (5). Here, we report the complete genome sequence of strain NSMJ15, which supports its characteristics as a potential probiotic candidate.

Strain NSMJ15 was first isolated by serial dilutions of a makgeolli suspension and plating onto MRS agar (Difco, USA) (5). For whole-genome sequencing, genomic DNA (gDNA) was extracted from strain NSMJ15 grown in MRS broth (Difco) at 30°C for 48 h using a Maxwell 16 DNA purification kit (Promega, USA). The gDNA was sequenced using the PacBio RS II platform and Illumina HiSeq X ten platform (2 × 151 bp) at Macrogen, Inc. (South Korea). Intact gDNA was sheared to approximately 20 kb using a g-TUBE device (Covaris, Inc., USA) and purified using AMPure PB magnetic beads, and a sequencing library was constructed using the PacBio SMRTbell template prep kit v1.0 (PacBio). For the Illumina sequencing, 100 ng gDNA was sheared using an LE220 focused ultrasonicator (Covaris, Inc.), and a sequencing library with a mean size of 350 bp was prepared using the TruSeq Nano DNA library prep kit (Illumina). A total of 50,575 subreads (0.60 Gbp; coverage, 202.53-fold; mean subread length, 11,845 bp; *N*_50_, 16,212 bp) generated using the PacBio RS II platform were used for *de novo* genome assembly using HGAP v3 (6). The overlap of both ends of each contig was checked to determine whether it was circular, and the overlapping ends were trimmed. A total of 5,902,004 quality-filtered Illumina paired-end reads (0.89 Gbp; coverage, 301.27-fold), of which ≥90% bases in each read had a Phred quality score of ≥30, were used for error correction with Pilon v1.21 to obtain the final genome assembly (7). Default parameters were used except where otherwise noted.

The final genome assembly had a mean sequencing depth of 105-fold and a GC content of 46.40%. It consisted of a 2,791,177-bp circular chromosome and four plasmids (pLPN-1, pLPN-2, pLPN-3, and pLPN-4). The genome assembly and annotation statistics are shown in Table 1. Average nucleotide identity (ANI) analysis was performed using OrthoANIu (8) to identify strain NSMJ15. It shared 98.34% sequence similarity with Lacticaseibacillus paracasei subsp. *paracasei* JCM8130^T^ (GenBank accession numbers AP012541 to AP012543). This value was higher than the ANI threshold range of 95 to 96% (9), indicating that strain NSMJ15 belongs to the species *Lacticaseibacillus paracasei*. Genome annotation using NCBI Prokaryotic Genome Annotation Pipeline (PGAP) v4.11 (10) identified 2,690 protein-coding genes, 15 rRNA genes, 60 tRNA genes, 3 noncoding RNAs (ncRNAs), and 179 pseudogenes. Data obtained from the RAST annotation server (11) showed that the genome contained 3,075 coding DNA sequences (CDSs) and 231 subsystems. Genes for metabolism of carbohydrates showed the highest prevalence (17.0%), followed by genes for protein metabolism (13.2%) and amino acid and derivatives (12.1%). Several cell surface proteins (classes A and C sortase), LPXTG-motif cell wall anchor domain proteins, and d-alanyl-lipoteichoic acid biosynthesis proteins (dltABCD) were detected in the strain NSMJ15 genome, which suggests its potential to adhere to intestinal epithelial cells (12). In particular, putative bacteriocin-encoding gene clusters were identified *in silico* using the BAGEL4 software tool (13), which revealed two areas of interest (AOIs) in the chromosome and four open reading frames (ORFs) encoding the core peptides of bacteriocin. Strain NSMJ15 also contained genes encoding ABC transporters and bacteriocin immunity for bacteriocin export and self-immunity to bacteriocin, respectively.

**TABLE 1:** Summary of assembly and annotation statistics for Lacticaseibacillus paracasei strain NSMJ15

Genetic element	Genome size (bp)	GC content (%)	No. of coding sequences	No. of rRNAs	No. of tRNAs	Sequencing depth (×)	GenBank accession no.
Chromosome	2,791,177	46.62	2,524	15	60	102	CP049324
pLPN-1	51,668	43.93	60	0	0	264	CP049325
pLPN-2	50,646	43.60	48	0	0	156	CP049326
pLPN-3	46,080	41.26	45	0	0	114	CP049327
pLPN-4	18,602	41.33	13	0	0	88	CP049328
Total	2,958,173	46.40	2,690	15	60	105	

### Data availability.

The genome sequence and raw sequencing reads for strain NSMJ15 were deposited under GenBank accession numbers CP049324, CP049325, CP049326, CP049327, and CP049328, BioProject accession number PRJNA607656, BioSample accession number SAMN14142699, and SRA accession numbers SRX9113284 and SRX9113285.
